# The Dining-Room

**Published:** 1859-11

**Authors:** 


### THE DINING-ROOM.

The New York *Spirit of the Times* aptly and beautifully says: "The refinement of a family is no where so quickly seen as at the table, and nowhere do men's sensual and selfish instincts become more prominent. There is the centre of a family after the day's wandering; there its first meeting after a night of forgetfulness; there we give hospitality to the stranger; there the tongue is loosened, the wandering thoughts called back, and the heart is warmed into expression, under generous fare."

We love to see and hear the cheerful greeting, as the members of the family, one by one, come to the breakfast table; there is politeness, refinement, elevation in it; and underlying all, a sweet affection, making the day begin with kindly courtesies, and the outgushing of warm hearts. Then, at the close of the day, its labors, its toils, its fears, its disappointments, and its dangers passed, to receive compensation for all, in rest and repose. The smile may be more languid, the voice and tone and gesture may be more subdued, but as pure an affection wells up from every heart, and as true a love speaks out from every eye, to soothe and soften the perturbations of the day; and at a later hour still, to bow around the family altar in humble, penitent, and loving devotion, in health and competence and safety! these are joys which kingdoms cannot purchase, but which are given to all who properly seek them. These are scenes which should be enacted every day, in every household in the land. But are they? And if not, why not? Let withering shame and crushing contempt be upon the father, mother, child, who stands in the way of happyfying scenes like these. How are the buds of joyousness blasted in many children's hearts, that would else have bloomed into flowers of gladness all day long, by a father's entrance to the breakfast room, after a night's debauch, whose first word is a stupid growl or coarser curse; or by the mother, whose shattered health, or broken ambition, or half-crazed intellect, or low nature, leads her, for successive weeks, and months, and years, with but an average exception in a score or more, to approach her gathered family always last, creeping in more dead than alive, with some fierce complaint, some low invective, some groundless charge, some frivilous objection, some mean insinuation, some reckless assertion, or some unendurable lie; or the pampered son or daughter, the pet of ignorance, of misplaced affection, or of senseless wealth, who comes to the table with swaggering contempt, or listless indifference, who by boisterous and imperious orders, or by inarticulate whispers, outrages the servants, insults the guests, and mortitifies the family—on all such mean, and selfish, and petulant natures, which scatter clouds where else there might be sunshine; which plant thorns where otherwise the sweetest flowers would have flourished—on people like these, we say, there will come, sooner or later, a fearful retribution of years without honor, of age without affection, and a lifetime wasted, if, indeed, it does not end in a premature death, or the more terrible mad-house.

